# Seismic response of mountain tunnel induced by fault slip

**DOI:** 10.1038/s41598-024-67225-0

**Published:** 2024-08-01

**Authors:** Chen Xia, Zian Wang, Yongsheng He, Yeqing Chen, Chunhai Li, Lei Shi, Chuan Zhao

**Affiliations:** 1 State Key Laboratory of Target Vulnerability Assessment, Institute of Defense Engineering, AMS, PLA, Beijing, China; 2 International Cooperation Base for Transportation Infrastructure Construction, Beijing University of Civil Engineering and Architecture, Beijing, China; 3 Quality Supervision Station, Joint Logistics Support Force Engineering, PLA, Nanjing, China

**Keywords:** Active fault, Tunnel, Nonuniform slip model, Discrete–continuous coupling analysis, Natural hazards, Civil engineering

## Abstract

With the rapid development of Chinese transportation networks, such as the Sichuan-Tibet railway, numerous tunnels are under construction or planned in mountainous regions. Some of these tunnels must traverse or be situated near active fault zones, which could suffer damage from fault slip. In this study, the seismic response of a mountain tunnel subjected to coseismic faulting was analyzed using a fault-structure system in a two-step process. Firstly, a nonuniform slip model was proposed to calculate the ground deformations and internal displacements induced by a specific active fault on a geological scale, considering nonuniform slips on the fault plane. The 1989 Loma Prieta and 2022 Menyuan earthquakes were chosen as case studies to validate the proposed slip model. Secondly, the calculated displacement of the Menyuan earthquake was used as the input load for the discrete–continuous coupling analysis of the Daliang tunnel on an engineering scale. The simulated deformation of the Daliang tunnel aligned with the on-site damage observations following the Menyuan earthquake. Lastly, the effects of different fault conditions on the tunnel seismic response were investigated. The results indicate that the distribution of the peak longitudinal strain of the lining is governed by fault mechanisms, and the degree of fault slip significantly influences the response of the tunnel. A tunnel passing through an active fault with a wider fault fracture zone and smaller dip angle experience less damage.

## Introduction

Earthquake impacts on underground structures can be categorized into two main effects: (1) ground shaking and (2) ground failure, such as fault slip^1^. The 1995 Kobe earthquake (Japan) caused a major collapse of the Daikai subway station in Kobe, which was the first modern shallow-buried underground structure to fail during a seismic event. According to Iida et al.^2^, the destructive horizontal force was created by the relative displacement between the base and ceiling levels due to subsoil movement. For most underground structures, the inertia of the surrounding soil is large relative to the inertia of the structure, which differs from the behavior of ground structures. Seismic design and analysis methods for underground structures have advanced rapidly since then, including laboratory model tests^3–5^, simplified analytical solutions^6–8^, and numerical modeling^9–11^.

With the rapid accumulation of near-fault ground motion records and observations, basic theories of seismology, physics of seismic sources, empirical relations of seismic sources, and scenario seismic event simulation methods have been widely studied and well developed. Seismological theories and methods can be applied to evaluate ground motions and deformations, which critically affect the seismic response of underground structures. Several studies have integrated the seismological theories with the structural seismic analysis using fault-structure simulation systems^12,13^. However, these studies mainly focus on the seismic response of structures to seismic waves.

In 1999, the Chi Chi earthquake significantly impacted several highway tunnels, including one through the Chelungpu fault, which was closed due to a 4-m fault slip^14^. Similar incidents have also been reported in Italy, the United States, and China^15–17^. To mitigate the potential threats of coseismic faulting, researchers and engineers have proposed various approaches using experimental model tests^18–20^ and numerical calculations^21,22^. The finite element and finite difference methods, based on continuum theory, have been widely used in numerical modeling due to their high computational efficiency. However, for discrete media or materials, such as fault fracture zones, ideal results for large deformation problems are challenging to achieve with continuum mechanisms. A discrete–continuous coupling method^23–25^ is more effective for seismic response analysis of tunnels passing through fault zones. According to Hashash et al.^1^, a systematic approach to evaluating the seismic response of underground structures involves three major steps: (1) defining the seismic environment and developing seismic parameters; (2) evaluating the ground response to shaking, including ground failure and deformations; and (3) assessing the structural behavior due to seismic shaking. The key challenge is to evaluate the seismic loads subjected to underground structures. In experimental model tests and numerical modeling, fault slip is usually assumed to be a constant, which is determined by empirical relationships^26^. However, fault plane slip is heterogeneous, causing spatially varied ground deformation^27–29^. Okada’s theories^30,31^ are widely used to obtain spatially varied ground deformation due to fault slip. although they assume constant slip on the fault, differing from real seismic events inversion.

In this study, the seismic response of a tunnel due to coseismic faulting is analyzed using a fault-structure system. First, a nonuniform fault slip model is proposed to estimate the surface and internal deformation in Section "Surface and internal deformation induced by nonuniform fault slip". The Loma Prieta and 2022 Menyuan earthquakes are selected to validate the nonuniform fault slip model. Then, the seismic response of the Daliang tunnel due to coseismic faulting is analyzed using discrete–continuous coupled numerical simulation in Section "Seismic response of the Daliang tunnel caused by spatial varied internal displacement". Finally, the effects of different fault conditions and parameters on the tunnel coseismic response of due to fault slip are discussed.

## Surface and internal deformation induced by nonuniform fault slip

To consider the spatially varied slip on the fault, a source model with a nonuniform slip distribution is proposed. The Loma Prieta and 2022 Menyuan earthquakes are selected as case studies to validate the proposed slip model.

### Method

**Figure 1:**
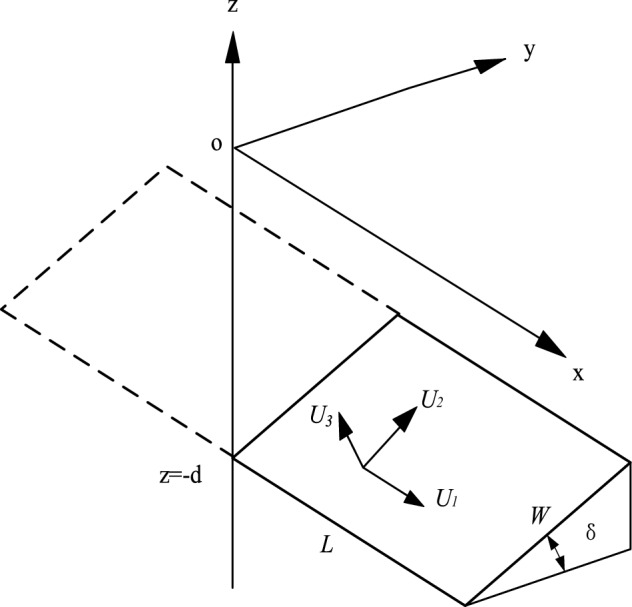
Geometry of the source model.

Okada^30,31^ proposed a comprehensive set of analytical expressions for surface and internal displacements, strains, and tilts owing to inclined shear and tensile faults in a half-space for both point and finite rectangular sources. As shown in Fig. 1, the x-direction is parallel to the strike of the fault, the y-direction is perpendicular to the strike, and the z-direction is vertical. The origin of coordinate O is located on the surface, which is the vertical projection of the lower left point on the footwall plane. δ, W, and L are the dip angle, width, and length of the fault plane, respectively. U_1_, U_2_, and U_3_ are the average dislocation components in the strike, dip, and tension directions, respectively. *λ* and *μ* are Lame’s constants of the underground medium. Surface displacement induced by fault slip can be determined by Okada’s solutions^30,31^.

In Okada’s methods^30,31^, the slip on the fault is assumed to be constant, and determined by empirical relations^26,32,33^. However, derived source models^24–27,27,28,28,29,29–36^ reveal that the slip distribution on the fault is highly heterogeneous. To consider the spatially varied slip in Okada’s methods, the fault plane is divided into several subfaults. Surface deformation induced by the subfault slip is calculated using Okada’s analytical solutions, and the total surface deformation is obtained by summing the contributions from each subfault.

### Uniform slip model of asperity

Seismologists define an asperity as a fault rupture region with higher slip relative to the average slip on the fault. Somerville et al.^27^ used a rectangular definition for asperities to facilitate slip model generation for future earthquakes. The average slip on the asperity is 2.01 times the slip averaged over the entire fault rupture surface.

**Figure 2:**
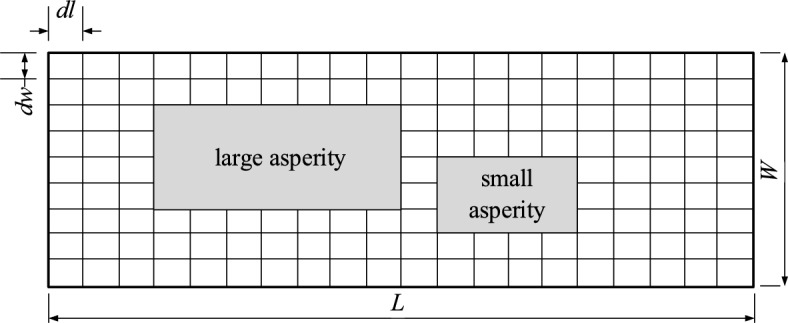
Asperity model with uniform slip. The gray areas represent the large and small asperities, respectively.

In the asperity model, the fault plane is divided into several subfaults of equal area, as shown in Fig. 2. *L* and *W* represent the length and width of the entire fault plane, respectively, while *dl* and *dw* are the length and width of the subfaults, respectively. For earthquake scenarios, the source parameters can be obtained as follows:

According to Aki and Richards^37^, the average slip $\overline{u}$ on the fault plane is determined by

1 $$ \overline{u}{ = }\frac{{M_{0} }}{\mu A}, $$

where $M_{0}$ denotes the seismic moment, $\mu$ denotes the shear modulus, and $A$ denotes the rupture area of the fault plane. The relationship between the seismic moment $M_{0}$ and the moment magnitude $M_{{\text{w}}}$ is^32^:

2 $$ M_{{\text{w}}} = \frac{2}{3}\left( {\log M_{0} - 9.1} \right), $$

and substituting Eq. (1) into Eq. (2) yields:

3 $$ \overline{u} = \frac{{10^{{1.5M_{{\text{w}}} + 9.1}} }}{\mu A}. $$

As shown in Eq. (3), the average slip of the fault is related to its moment magnitude, shear modulus, and rupture area. In Wells and Coppersmith’ empirical relations^26^, the fault rupture area and fault length are related to the seismic magnitude as follows:

4 $$ \log A = - 3.49 + 0.91M_{{\text{w}}} , $$

5 $$ \log L = - 2.44 + 0.59M_{{\text{w}}} . $$

Subsequently, the traditional asperity model with uniform slip can be determined using the following procedures^27^:

1. Two asperities are set on the entire fault plane: one large and one small.
2. The areas of the large and small asperities are $A_{as1} = 0.16LW$ and $A_{as2} = 0.06LW$, respectively.
3. The average slip of the asperities $\overline{u}_{as}$ is approximately 2.01 times the value of the entire fault plane slip $\overline{u}_{as} { = }2.01\overline{u}$.
4. The average slip of the background area $\overline{u}_{b}$ is approximately 0.71 times the value of the entire fault plane slip $\overline{u}_{b} { = 0}{\text{.7}}1\overline{u}$.

The surface and internal deformation induced by the slip of subfaults in the asperity and background regions can be calculated using Okada’s theories^30,31^. The total seismic moment of the asperities and background area equals the seismic moment of the target fault:

6 $$ M_{0} = M_{{0{\text{as}}1}} + M_{{0{\text{as}}2}} + M_{{0{\text{b}}}} , $$

where $M_{0}$,$M_{{0{\text{as}}1}}$,$M_{{0{\text{as}}2}}$, and $M_{{0{\text{b}}}}$ denote the seismic moments of the entire fault, large asperity, small asperity, and background, respectively.

### Nonuniform source slip model

The definition of asperities can consider slip variation over the entire fault plane. However, slip variation within the asperity region cannot be account for. Moreover, the slips on the edge of the asperity and the adjacent background area exhibit a dramatic change in the uniform asperity slip model, which does not reflect reality. Derived source slip models indicate that the slip on the asperity gradually decreases from the central zone to the boundary.

**Figure 3:**
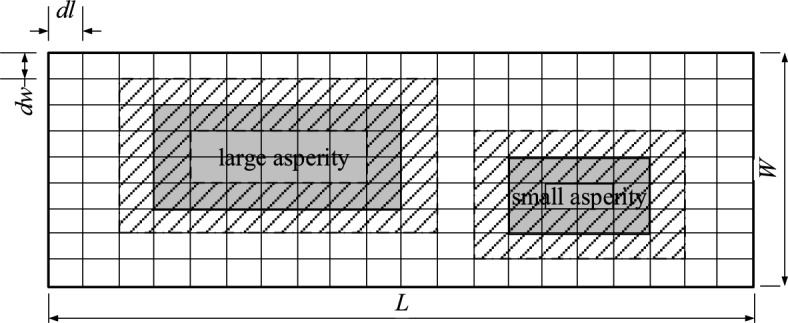
Asperity model with nonuniform slip. The gray area filled with lines represents subfaults on the asperity adjacent to the background area, and the white area filled with lines represents subfaults in the background area adjacent to the asperity.

To address the spatial variation of the slip on the asperity, a nonuniform asperity slip model is proposed, in which the slip on the asperity gradually changes, as shown in Fig. 3. The gray area filled with lines represents subfaults on the asperity adjacent to the background area, and the white area filled with lines represents subfaults in the background area adjacent to the asperity. There are two asperities in this model: a large one and a small one. For a fault with *n* subfaults of the same size, when the average slip $\overline{u}_{as}$ of the asperities and the average slip $\overline{u}_{b}$ of the background on the fault have been obtained, the dislocation amount of subfaults can be determined by the following method.

For an asperity with *n*_as_ subfaults, there are *n*_aso_ subfaults adjacent to the background area, with an average dislocation $\overline{u}_{{{\text{aso}}}}$, and *n*_asi_ subfaults not adjacent to the background region, with an average dislocation $\overline{u}_{{{\text{asi}}}}$. $\overline{u}_{{{\text{aso}}}}$ and $\overline{u}_{{{\text{asi}}}}$ can be determined as follows:

7 $$ n_{{{\text{as}}}} \mu \overline{u}_{{{\text{as}}}} A_{{{\text{sub}}}} { = }n_{{{\text{aso}}}} \mu_{1} \overline{u}_{{{\text{aso}}}} A_{{{\text{sub}}}} + n_{{{\text{asi}}}} \mu_{2} \overline{u}_{{{\text{asi}}}} A_{{{\text{sub}}}} . $$

Assuming the shear modulus of the entire fault plane is constant, Eq. (7) takes the following form:

8 $$ n_{{{\text{as}}}} \overline{u}_{{{\text{as}}}} { = }n_{{{\text{aso}}}} \overline{u}_{{{\text{aso}}}} + n_{{{\text{asi}}}} \overline{u}_{{{\text{asi}}}} . $$

Moreover, the average slip $\overline{u}_{{{\text{asi}}}}$ is assumed to be two times of $\overline{u}_{{{\text{aso}}}}$

9 $$ \overline{u}_{{{\text{aso}}}} = 0.5\overline{u}_{{{\text{asi}}}} . $$

The dislocation of each subfault in the adjacent background area on the asperity $\overline{u}_{{{\text{aso}}i}}$($1 \le i \le n_{{{\text{aso}}}}$) and the dislocation of the other subfaults on the asperity $\overline{u}_{{{\text{asi}}j}}$($1 \le j \le n_{{{\text{asi}}}}$) have the following relationships:

10 $$ \sum\limits_{i = 1}^{{n_{{{\text{aso}}}} }} {\mu A_{{{\text{sub}}}} \overline{u}_{{{\text{aso}}i}} = n_{{{\text{aso}}}} \mu A_{{{\text{sub}}}} \overline{u}_{{{\text{aso}}}} } , $$

11 $$ \sum\limits_{j = 1}^{{n_{{{\text{asi}}}} }} {\mu A_{{{\text{sub}}}} \overline{u}_{{{\text{asi}}j}} = n_{{{\text{asi}}}} \mu A_{{{\text{sub}}}} \overline{u}_{{{\text{asi}}}} } , $$

Since the shear modulus of the entire fault plane is constant, and the subfaults have the same areas, Eqs. (10) and (11) can be rewritten as follows:

12 $$ \sum\limits_{i = 1}^{{n_{{{\text{aso}}}} }} {\overline{u}_{{{\text{aso}}i}} = n_{{{\text{aso}}}} \overline{u}_{{{\text{aso}}}} } , $$

13 $$ \sum\limits_{j = 1}^{{n_{{{\text{asi}}}} }} {\overline{u}_{{{\text{asi}}j}} = n_{{{\text{as}}j}} \overline{u}_{{{\text{as}}j}} } , $$

To account for the variation in $\overline{u}_{{{\text{aso}}i}}$, $\overline{u}_{{{\text{aso}}i}}$ is assumed to take a random value in the range between $0.5\overline{u}_{{{\text{aso}}}}$ and $1.5\overline{u}_{{{\text{aso}}}}$. $\overline{u}_{{{\text{asi}}j}}$ follows the same rule. The slip on the asperity can then be determined by this assumption and Eqs. (8), (9), (12), and (13). The slip on the background area can be determined using the same method. Using the above method, the nonuniform slip on the source can be obtained, which considers the general characteristics of the inversed source models.

### Surface deformation of Loma Prieta earthquake by nonuniform slip model

**Figure 4:**
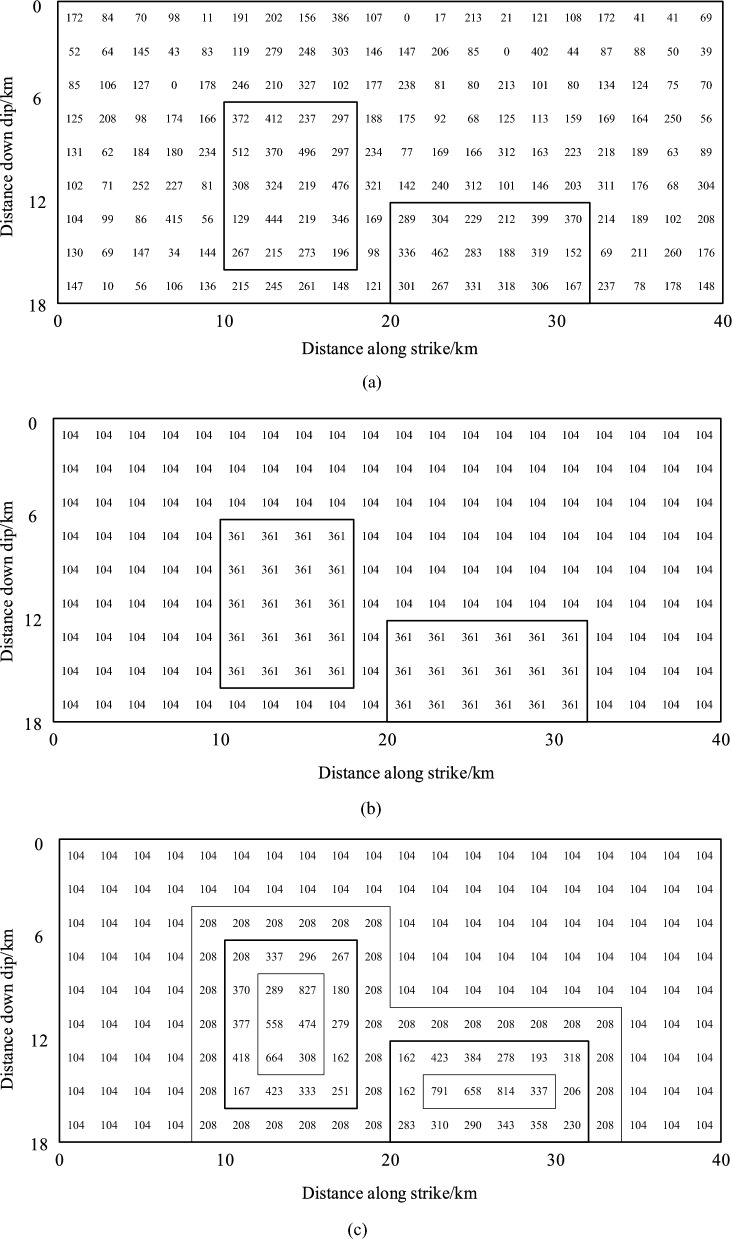
Three different slip model of the Loma Prieta earthquake (unit: cm), (**a**) Model 1: slip model of the Loma Prieta earthquake inverted by Somerville et al.^27^, (**b**) Model 2: uniform asperity model of the Loma Prieta earthquake, and (**c**) Model 3: nonuniform asperity model of the Loma Prieta earthquake.

In this section, the 1989 *M*_w_6.95 Loma Prieta earthquake is selected as the target event. The main source parameters of the Loma Prieta earthquake are presented in Table 1. To validate the proposed nonuniform slip model, three different slip distribution models are constructed as shown in Fig. 4. All three source models have the same seismic moments. Model 1 is an inverted slip distribution based on seismic data^27^. In Model 2, the dislocations on the asperities and the background are uniform. In Model 3, the heterogeneous slip distribution is determined using the method proposed in Section "Nonuniform source slip model". The surface deformations of the 1989 Loma Prieta earthquake are calculated using these three models.

**Table 1:** Source parameters of the Loma Prieta earthquake.

Parameter	Value
Length (km)	40
Width (km)	18
Depth (km)	3.4
Strike (°)	128
Dip (°)	70
Slip (°)	135

**Figure 5:**
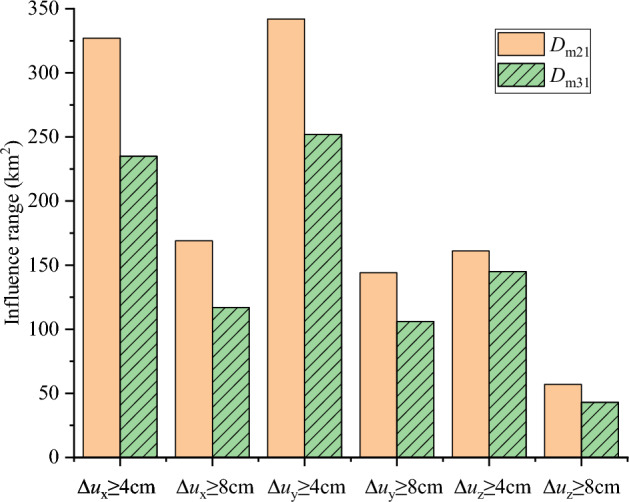
Histogram of the influence range of displacement residual between different dislocation modes and the real situation. Areas with the surface deformation difference larger than 0.04 m and larger than 0.08 m. The yellow bars and green bars with dash line represent the results of *D*_m21_ and *D*_m31_, respectively. $\Delta u_{{\text{x}}}$, $\Delta u_{{\text{y}}}$, and $\Delta u_{{\text{z}}}$ represent the surface difference in three different directions, respectively.

The surface deformation calculated using Model 1 is used as the reference. The surface deformation difference between Models 2 and 1 is denoted as *D*_m21_, and *D*_m31_ follows the same rule. Figs. S1 and S2 display the surface displacement differences *D*_m21_ and *D*_m31_ in three directions, respectively. The thick gray solid line is the fault line, and the origin of the coordinates is located at the center point of the fault line. The surface deformation difference *D*_m31_ is smaller than *D*_m21_, indicating that the surface deformation calculated by Model 3 is closer to the result of Model 1. The areas with the surface deformation difference larger than 0.04 m and larger than 0.08 m are compared in Fig. 5. The yellow bars represent the results for *D*_m21_, and the green bars with dashed lines represent the results for *D*_m31_. The areas of surface deformation difference are compared in three directions, which are represented as $\Delta u_{{\text{x}}}$, $\Delta u_{{\text{y}}}$, and $\Delta u_{{\text{z}}}$. As shown in Fig. 5, the values of the yellow bars are all larger than those of the green bars, indicating that Model 3 with nonuniform asperity slip achieves better performance than Model 2 with uniform asperity slip.

### Surface deformation of Menyuan Ms6.9 earthquake

On January 8, 2022, a magnitude 6.9 earthquake occurred in Menyuan County, Qinghai Province. The hypocenter of the Menyuan *M*_s_6.9 earthquake was located at 37.77°N, 101.26°E, with a depth of 10 km. This event injured several people and caused damage of infrastructure. The main source parameters of the Menyuan *M*_s_6.9 earthquake are summarized in Table 2.

**Table 2:** Source parameters of the *M*_S_6.9 Menyuan earthquake.

Parameter	Value
Length (km)	25
Width (km)	10
Depth (km)	10
Strike (°)	289
Dip (°)	88
Slip (°)	0

**Figure 6:**
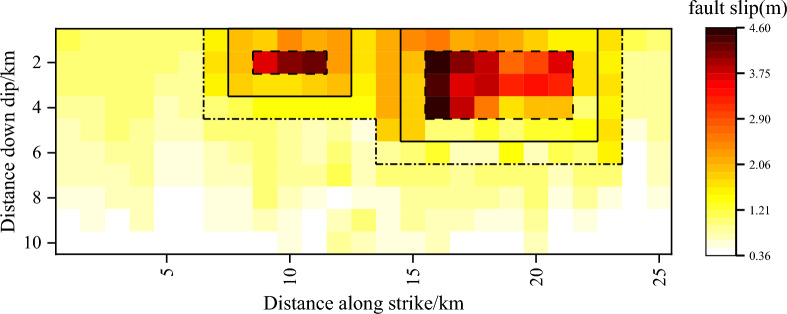
Nonuniform asperity dislocation model of the Menyuan MS6.9 earthquake.

The inverted dislocation distribution on the source plane is shown in Fig. S3 (https://earthquake.usgs.gov/). Fig. S4 is the inverted slip model of Menyuan earthquake by Zhang et al.^38^. And the heterogeneous slip model inverted by Zhang et al.^38^ is also referenced to build up the proposed nonuniform slip model, as shown in Fig. 6.

**Figure 7:**
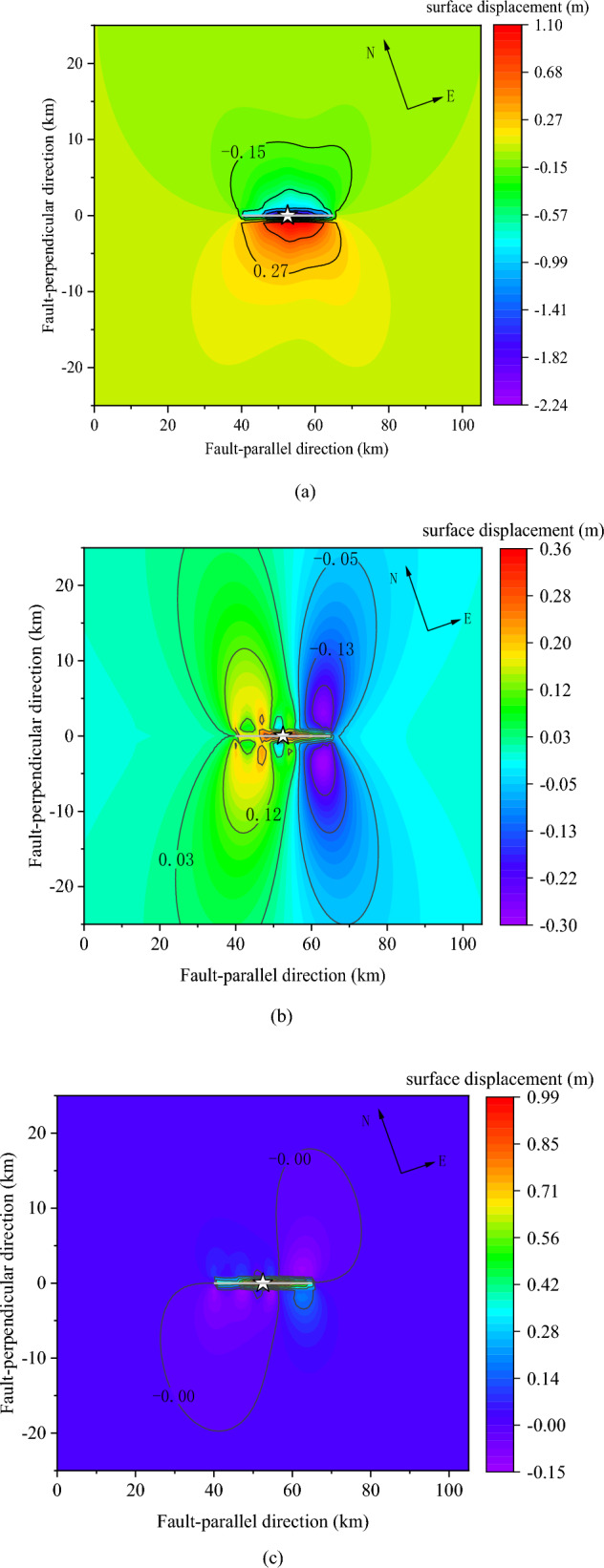
Surface displacement induced by the Menyuan *M*_S_6.9 earthquake fault (unit: m), (**a**) horizontal surface displacement difference in the fault-parallel direction, (**b**) horizontal surface displacement difference in the fault-perpendicular direction, and (**c**) surface displacement difference in the vertical direction. The thick gray lines represent the fault lines.

Figure 7 shows the surface deformation induced by the Menyuan *M*_s_6.9 earthquake, which is calculated based on the source slip model in Fig. 6. The thick gray line represents the fault line, and the star marks the epicenter.

In the fault-parallel direction (Fig. 7a), the displacement is positive in the southeast direction and negative in the northwest direction. The surface deformation shows a symmetrical distribution along the fault line but in opposite directions. In the near-fault region, the average surface deformation in the fault-parallel direction is larger than 1.5 m, with a maximum value of 2.24 m near the epicenter. The surface deformation decreases to 0.15 m at a fault distance of 10 km. According to the field research by Institute of Geology, China Earthquake Administrator(https://eq-igl.ac.cn/zhxw/info/2022/36632.html), the maximum dislocation is over 2 m. The results of inverted surface deformation are matches the field research. Based on the inverted surface deformation of the Liuhuangou Bridge near the Daliang tunnel conducted by Liu et al.^39^, our results also show a close correlation with their findings.

In the fault-perpendicular direction (Fig. 7b), the surface displacement is negative in the northeast direction and displays a symmetric distribution. The maximum surface deformation is 0.36 m at a site with a fault distance of 1.5 km. Near the epicenter, the surface deformation is approximately 0 m. The vertical surface deformation (Fig. 7c) is remarkably smaller than that in the horizontal direction and shows a centrosymmetric distribution along the fault line. The maximum uplift deformation is 0.99 m, and the maximum surface subsidence is 0.15 m near the fault. Surface deformation in the three directions corresponds to the general characteristics of a strike-slip fault.

## Seismic response of the Daliang tunnel caused by spatial varied internal displacement

The earthquake damage investigation after the 2022 *M*_s_6.9 Menyuan earthquake shows that the Daliang tunnel of the Lanxin railway was damaged due to fault slip. In this section, the response of the Daliang Tunnel is estimated using a continuous-discrete coupling numerical simulation method. FLAC^3D^ and PFC^3D^ programs are used to perform the discrete–continuous coupling simulations. The spatially varied surface deformation calculated in Section "Surface deformation of Menyuan Ms6.9 earthquake" is used as the input seismic load.

### Description of the Daliang tunnel and discrete–continuous coupling model

**Figure 8:**
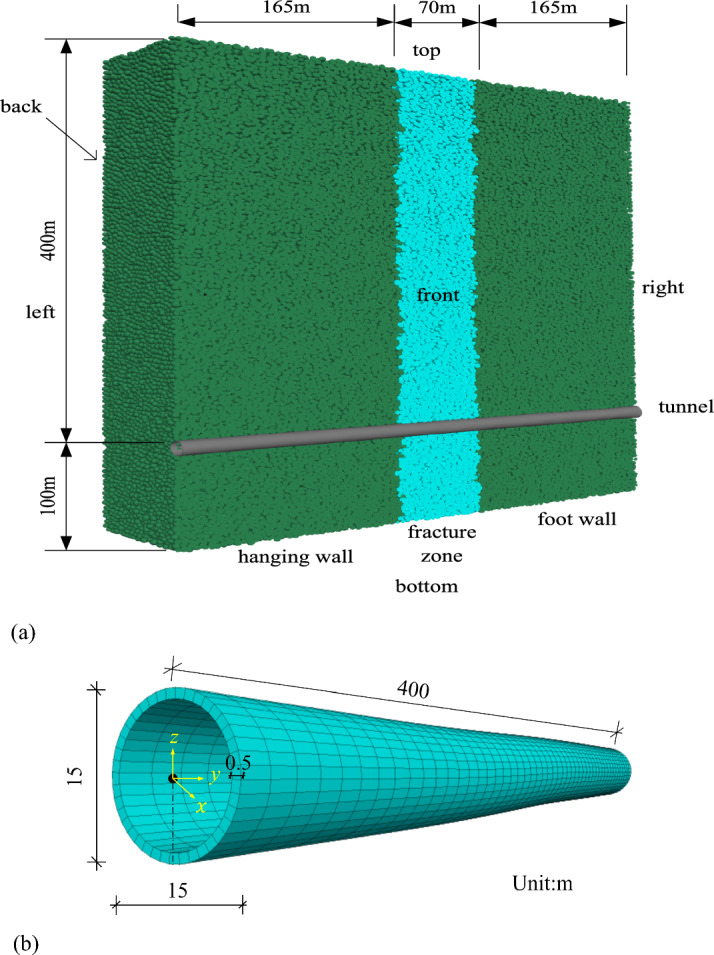
Numerical model of Daliang tunnel and surrounding rocks, (**a**) coupled discrete–continuous model of the Daliang tunnel and surrounding rock mass and (**b**) model of the Daliang tunnel.

The dip angle of the fault was 88°(https://earthquake.usgs.gov/) and the width of the fault fracture zone was 70 m. The discrete–continuous coupling model with surrounding rocks and a fault zone is 200 m wide, 500 m high, and 400 m in the longitudinal direction. The coupled discrete–continuous model of the Daliang tunnel and surrounding rock mass is shown in Fig. 8a. The Daliang tunnel has a radius of 7.5 m, a lining of 0.5 m, a longitudinal length of 400 m, and a depth of 400 m. The dimensions of the tunnel are detailed in Fig. 8b. The boundary conditions of the model involve applying the internal deformation calculated in Section "Surface deformation of Menyuan Ms6.9 earthquake" to the top, bottom, left, and right boundaries of the hanging wall, as well as to part of the fracture zone. The rest of the model are fixed to ensure stability.

FLAC^3D^ and PFC^3D^ programs are used to perform the discrete–continuous coupling simulations. The surrounding rock and fault fracture zone are modeled as discrete particles, and the parameters of the discrete particles are listed in Table 3. The tunnel structure is modeled as continuous elements, the behavior of which is described by the Mohr–Coulomb plastic model. The tunnel parameters are listed in Table 4. These parameters are determined based on the research of Zhang et al.^40^. The coupling process between the discrete and continuous elements is illustrated in Fig. S5, which is described by Cai et al.^41^ and Ma et al.^25^. The internal deformation calculated in Section "Surface deformation of Menyuan Ms6.9 earthquake" is employed as the input seismic load.

**Table 3:** Parameters of discrete particles.

Ball group	Density (kg/m^3^)	Friction coefficient	Damp	Cohesion force (MPa)	Friction angle (°)	Normal/tangential stiffness ratio
Surrounding rock	2300	0.3	0.7	0.25	30	1
Fault fracture zone	2300	0.3	0.7	0.1	25	1

**Table 4:** Parameters of tunnel.

Constitutive model	Density (kg/m^3^)	Young's modulus (GPa)	Poisson's ratio
Mohr–Coulomb	2600	20	0.25

### Response of the Daliang tunnel by discrete–continuous coupled numerical simulation

**Figure 9:**
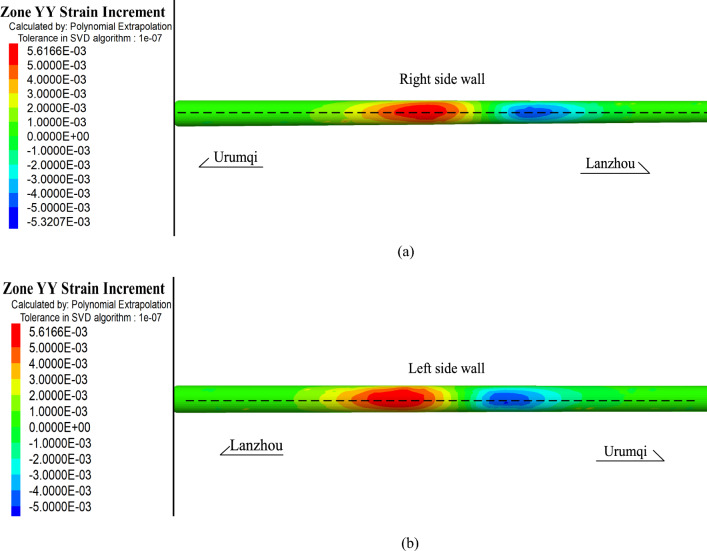
Longitudinal strain of the Daliang tunnel, (**a**) right side wall and (**b**) left side wall.

Figure 9 shows the longitudinal strain of the lining, which is considered positive during tensile deformation. The region with a large longitudinal strain of the lining is concentrated around the fault zone within a range of 40 m. The maximum tensile strain of the right side wall exceeded 5.5 × 10^3^ με, and the maximum tensile strain of the left side wall is approximately 5.5 × 10^3^ με. The local deformation in the lining has a range of 150 m in the fault zone. According to the elastic modulus of the material, the ultimate compressive strain and tensile strain of the material are 3 × 10^3^με and 1 × 10^2^με, respectively.

**Figure 10:**
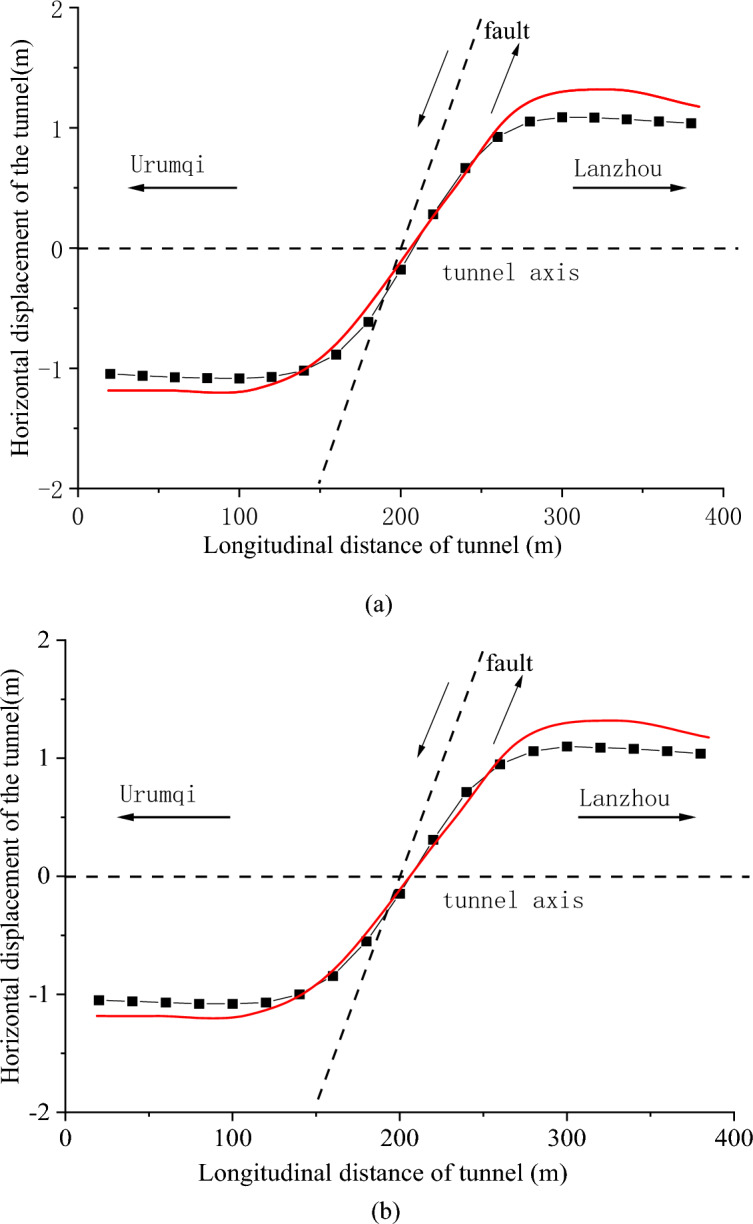
Horizontal deformation of the Daliang tunnel, (**a**) right side wall and (**b**) left side wall. The red solid curves are the schematic diagram of the real deformation of the tunnel.

The horizontal displacements of the left and right side walls are shown in Fig. 10a and b, respectively. The northward displacement of the tunnel is taken as positive, and the maximum relative dislocation of the tunnel is approximately 1.09 m. Because the fault is set as a left-lateral strike-slip fault in the numerical model, there is no obvious vertical deformation of the tunnel. The maximum uplift and maximum subsidence of the tunnel are 0.1 m and 0.2 m, respectively.

On-site seismic damage investigation shows that the tunnel is severely dislocated in the fault zone by Zhang et al.^38^ and the Institute of Geology, China Earthquake Administrator(https://eq-igl.ac.cn/zhxw/info/2022/36632.html). The maximum horizontal dislocation of the Daliang tunnel are over 2.5 m, respectively. The rail exhibits an S-shaped deformation, which is represented by the red curve in Fig. 15. The maximum extruded dislocation of the left side wall and right side wall are 2.16 m and 2.17 m, respectively. The observed results satisfy the characteristics of left-lateral strike-slip faults.

The response of the Daliang tunnel calculated by discrete–continuous coupled numerical simulation is generally consistent with the on-site seismic damage investigation. However, the maximum dislocation in the numerical model is smaller than the observed result, particularly in the vertical direction. This discrepancy can be attributed to two reasons. The first is the difference between the parameters in the numerical model and the real surrounding rocks in both the internal deformation simulation and the response analysis of the tunnel. The second reason is the effect of seismic waves on the tunnel, which is not considered in the numerical model. During an earthquake, both seismic waves and dislocations affect the response of the structures. In this section, we focus only on the response induced by ground deformation, which causes an underestimation of the deformation of the tunnel after an earthquake. Despite the above-mentioned discrepancy, the regional and overall stress and deformation characteristics of the tunnel model satisfy the real status of the Daliang tunnel after the *M*_s_6.9 Menyuan earthquake. The numerical simulation results show that the discrete–continuous coupling numerical model is valid for the analysis of tunnel passing through fault zones.

## Discussion-effects of different fault conditions on the response of tunnels

Different active faults have varying parameters that cause different ground deformation distributions. To investigate the effects of different fault parameters on the response of tunnels through active faults, a discrete–continuous coupling numerical simulation is performed under different fault mechanisms, fault slips, rupture fault widths, fault dip angles, and location of the asperity.

### Effect of the fault mechanism on the tunnel

**Figure 11:**
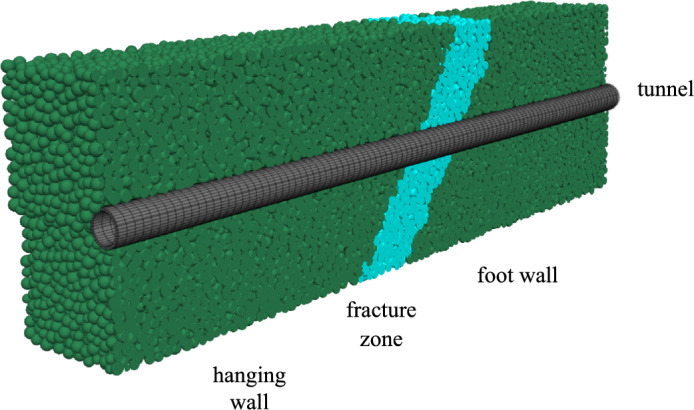
Discrete–continuous coupling numerical model of the strike-slip fault and tunnel.

**Figure 12:**
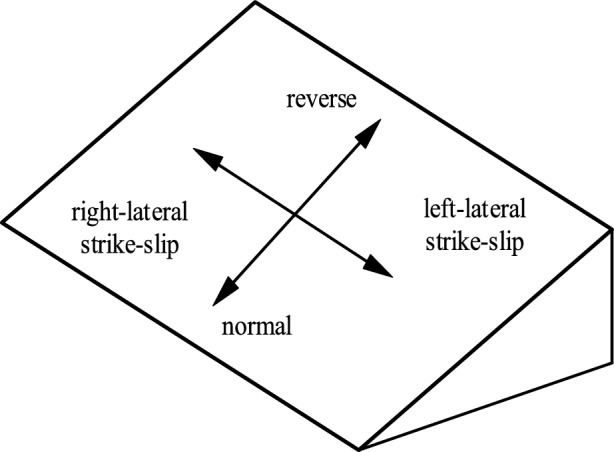
Schematic diagram of the fault mechanism. The pointer represents the direction of movement of the hanging wall.

Different earthquakes may be caused by different fault mechanisms, such as normal, reverse, and strike-slip faults. To investigate the effect of the fault mechanism on the seismic response of the tunnels, four different fault mechanisms are considered: normal, reverse, left-lateral strike-slip, and right-lateral strike-slip faults. The discrete–continuous coupling model is 60 m wide, 60 m high, and 200 m in the longitudinal direction. The fracture zone is 20 m wide, and the dislocation of the fault is 0.6 m. The dip angle of the strike-slip faults is 60°. The cross-section of the tunnel has a diameter of 8 m and a thickness of 0.5 m. The buried depth of the tunnel is 26 m, and its calculated length is 200 m. The other parameters of the tunnel, surrounding rock, and fault fracture zone are the same as those in Section "Description of the Daliang tunnel and discrete–continuous coupling model", as listed in Table 3 and Table 4. The numerical model of the strike-slip fault and tunnel is shown in Fig. 11. The models of other three fault mechanisms are the same as that of strike-slip fault, the only difference is the slip direction. Figure 12 shows the different fault mechanism.

**Figure 13:**
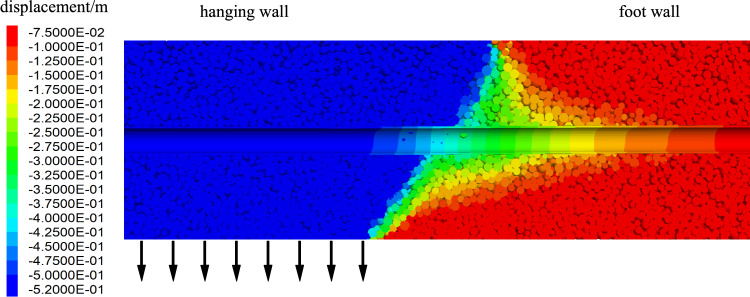
Vertical displacement of coupling model.

Section "Description of the Daliang tunnel and discrete–continuous coupling model" elucidates the principles of force and velocity transfer at the interface between FLAC3D and PFC3D during coupling, where contact forces are linked to displacements through stiffness in the contact surfaces. To validate the effectiveness of the coupled model, a normal fault model with a 60° dip angle was employed, examining displacements in both discrete and continuous elements. The model parameters include a 0.6 m fault slip and a 20 m fracture zone width, as depicted in Fig. 11. The resulting vertical displacement of the tunnel crossing the fault is illustrated in Fig. 13.

The displacement contour map shows that the fault dip induces approximately 0.52 m of vertical displacement in the hanging wall with a 0.6 m fault slip. The tunnel moves downward along with the fault and deforms near the fracture zone. The uniform displacement scale for the fault and tunnel in the map indicates strong continuity between the fault's discrete particles and the tunnel's continuous structure.

**Figure 14:**
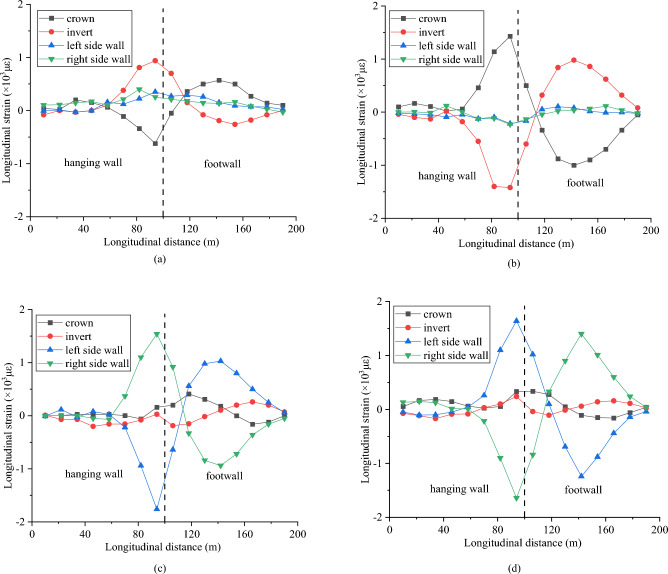
Longitudinal strain curve of tunnel under different fault slip mechanisms, (**a**) normal fault, (**b**) reverse fault, (**c**) left-lateral strike-slip fault, and (**d**) right-lateral strike-slip fault.

Figure 14 displays the longitudinal strain of the crown, invert, left side wall, and right side wall under four different fault mechanisms. For the normal and reverse faults, the peak longitudinal strains of the tunnel appear at the crown and invert. For the strike-slip faults, the peak longitudinal strains of the tunnel appear at the left side and right side walls. These results indicate that the distribution of the peak strain in the tunnel is governed by the fault mechanism. For tunnels passing through active fault zones, the fault mechanism should be considered in the seismic design of tunnels. The peak deformation in the tunnel is mainly concentrated in the vicinity of the fault fracture zone. In these cases, the tensile strain in the tensile part of the tunnel exceeds the ultimate tensile strain of the concrete, indicating that the damage to the tunnel is caused by tensile deformation.

### Effect of the fault slip on the tunnel

The fault slip increases with the earthquake magnitude. To investigate the effect of fault slips on the response of the tunnel through active faults, the fault slip is set to be 0.2 m, 0.6 m, 0.8 m, and 1 m. The fault is assumed to be a reverse fault with a dip angle of 60° and a fracture zone width of 20 m. The discrete–continuous coupling model is 60 m wide, 60 m high, and 200 m in the longitudinal direction. The buried depth of the tunnel is 26 m, and its calculated length is 200 m. The parameters of the tunnel, surrounding rock, and fault fracture zone are the same as those in Section "Description of the Daliang tunnel and discrete–continuous coupling model", as listed in Tables 3 and 4.

**Figure 15:**
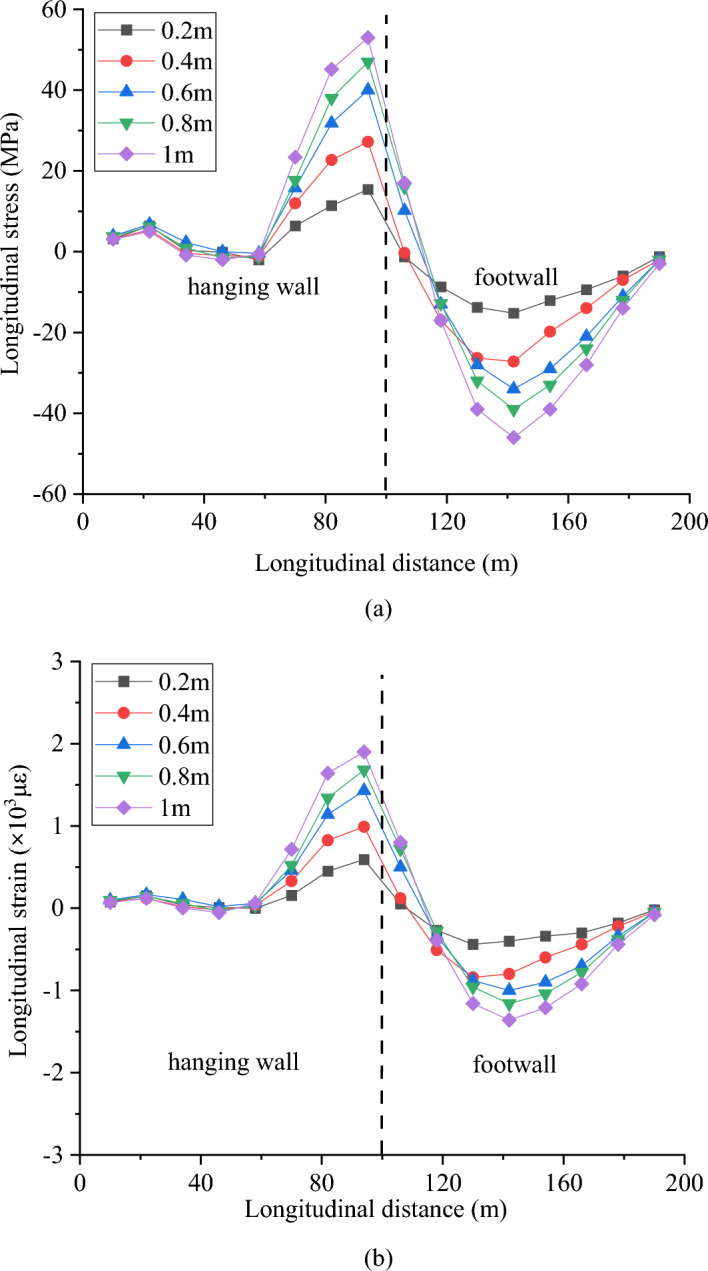
Response of the crown under different fault slips, (**a**) longitudinal stress and (**b**) longitudinal strain.

Figures 15a and b show the longitudinal stress and strain of the crown under different fault slips. The stress and strain of the tunnel are considered to be positive during tensile deformation. The longitudinal stress and strain generally increase with the fault slip. The maximum longitudinal tensile stress at the crown appears on the hanging wall, approximately 10 m from the center of the fault fracture zone. The maximum longitudinal compressive stress occurs on the footwall, at a distance of 40 m from the center of the fault fracture zone. The compressive area of the crown is larger than the tensile area. When the slip of the fault reaches 0.4 m, the maximum tensile stress of the tunnel crown exceeds 20 MPa. In the longitudinal strain curve, the tensile strain is positive and the compressive strain is negative. The strain curves display similar trends to the stress curves. When the fault slip reaches 0.4 m, the maximum tensile strain of the tunnel exceeds 1 × 10^3^με, which obviously reached the ultimate tensile strain of concrete, while the compression area is relatively safe.

### Effect of the rupture fault width on the tunnel

According to a previous report, underground structures would experience more serious damage in regions with varied geological conditions^42^. The fault fracture zone has different geological parameters and characteristics from those of the surrounding rock, which may affect the response of the tunnel through the fault zones. In this section, the width of the fault fracture zone is set to 10 m, 20 m, 30 m, and 40 m, respectively. The fault is assumed to be a reverse fault with a dip angle of 60°. The discrete–continuous coupling model is 60 m wide, 60 m high, and 200 m in the longitudinal direction. The buried depth of the tunnel is 26 m, and its calculated length is 200 m. The dislocation of the fault is 0.6 m. The other parameters of the tunnel, surrounding rock, and fault fracture zone are the same as those in Section "Description of the Daliang tunnel and discrete–continuous coupling model", as listed in Table 3.

**Figure 16:**
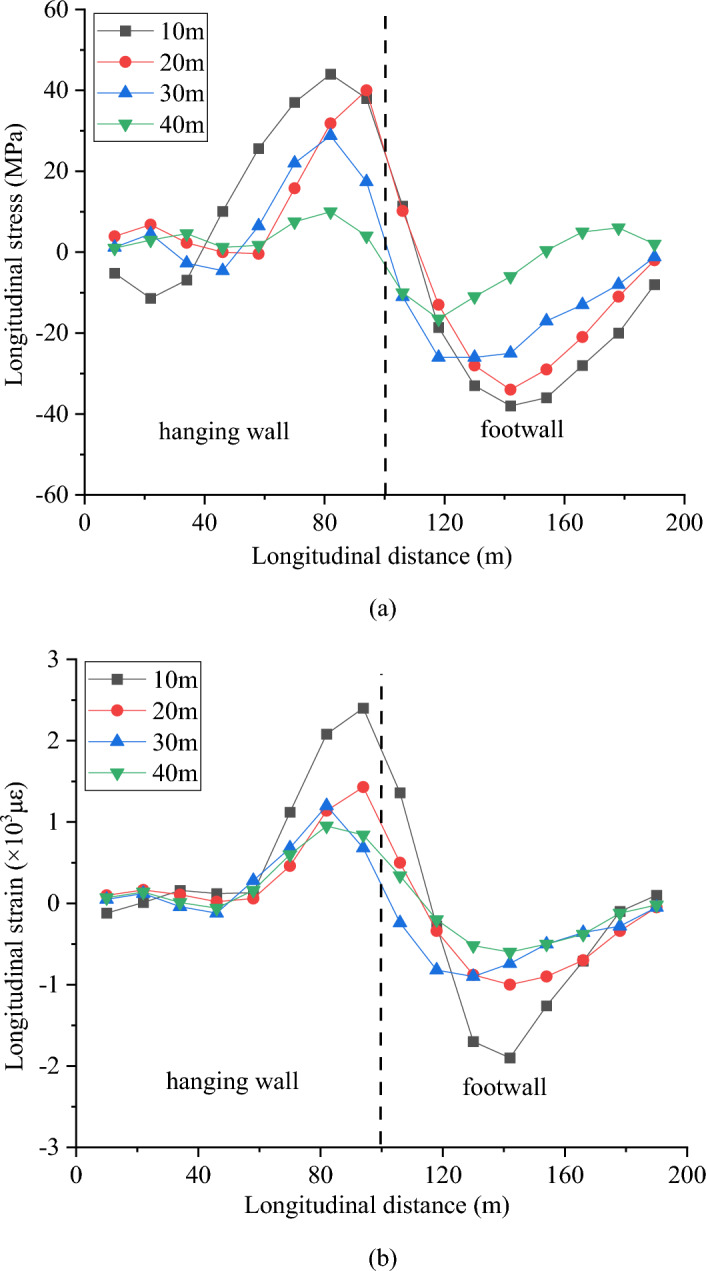
Response of the crown under different fracture zone widths, (**a**) longitudinal stress and (**b**) longitudinal strain.

Figure 16 shows the longitudinal stress and strain at the crown for different widths of the fault fracture zones. The maximum longitudinal stress and strain decrease as the width of the fault fracture zone increase. The maximum longitudinal tensile stress at the crown appears on the hanging wall, approximately 20 m from the center of the fault fracture zone. The maximum longitudinal compressive stress occurs on the footwall, at a distance of 40 m from the center of the fault fracture zone. Compared with the case of the 40 m wide fault fracture zone, the maximum tensile stress of the crown in the 10 m wide fault zone increases by 340%, and the maximum compressive stress increases by approximately 130%. The strain exhibits a similar change. The strength of the fault fracture zone is generally lower than that of the surrounding rock, which causes damage to the tunnel concentrated in this region. With a larger fault fracture zone width, the tunnel damage would be distributed in a larger area, but the maximum values of the stress and strain would be smaller.

### Effect of the dip angle on the tunnel

In this section, the dip angle of the fault is set to 30°, 45°, and 60° to investigate the effect of the dip angle on the response of the tunnel. The fault is assumed to be a reverse fault with a fracture zone width of 20 m. The discrete–continuous coupling model has the same size as that in Section "Description of the Daliang tunnel and discrete–continuous coupling model". The buried depth of the tunnel is 26 m, and its calculated length is 200 m. The dislocation of the fault is 0.6 m. The other parameters of the tunnel, surrounding rock, and fault fracture zone are the same as those in Sect. 3.1, as listed in Tables 3 and 4.

**Figure 17:**
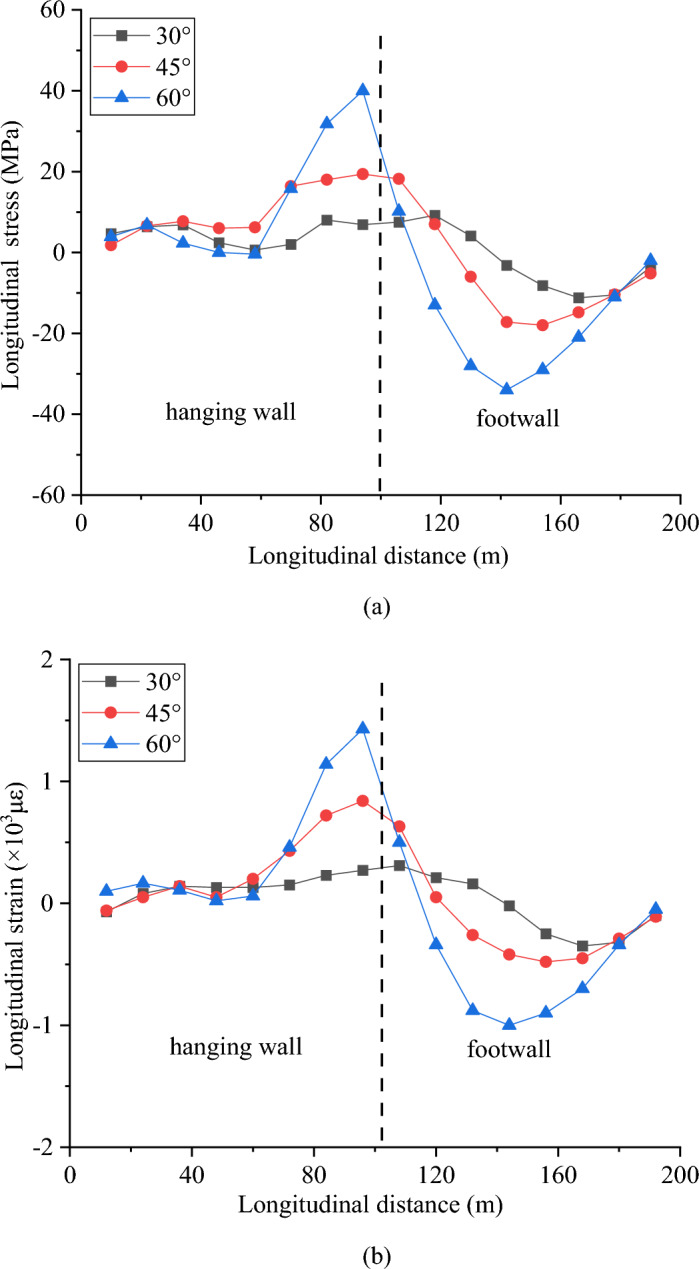
Response of the crown under different fault dips, (**a**) longitudinal stress and (**b**) longitudinal strain.

Figure 17 shows the longitudinal stress and strain at the crown under different dip angles. As the fault dip angle increases from 30° to 60°, the maximum stress and strain of the crown gradually increase. Because the fault is a reverse fault and the fault slip is constant, as the dip angle of the fault increases, the vertical component of the fault slip increases, resulting in stronger compression of the tunnel crown and invert. The horizontal component of the dislocation has little effect on the longitudinal stress of the tunnel; therefore, under the condition of the same fault slip, a larger fault dip angle will have a more adverse effect on the tunnel.

The above numerical simulation results indicate that fault slips can significantly affect the response of the tunnel. A tunnel through an active fault with a wider fault fracture zone and smaller dip angle would experience less damage. Moreover, the distributions of the maximum stress and strain change with different fault conditions.

### Effect of the location of the asperity on the tunnel

The location of the rupture initiation point is a critical parameter in simulating ground motion, as it influences both the time delay and the rupture propagation direction, thereby significantly impacting the simulated ground motion of the scenario earthquake^43,44^. Ground surface displacement simulations are based on Okada’s models, which do not incorporate temporal factors. Consequently, while the rupture initiation point profoundly affects the ground motion of the scenario earthquake, its impact on surface displacement is minimal.

In this section, the influence of the location of the asperity on the tunnel’s response is investigated. The properties of the Menyuan earthquake are used to develop the nonuniform asperity model. We adjust the asperity's location and propose three different source models, which are displayed in Fig. S6. Subsequently, the ground deformation for each source model is calculated, shown in Fig. S7 with the x dislocation along the fault. As the large asperity shifts to the right, the peak value of the x dislocation also moves rightward. This deformation is then applied to the tunnel model to study its influence on the tunnel structure. The fault is modeled as a left-lateral strike-slip fault.

**Figure 18:**
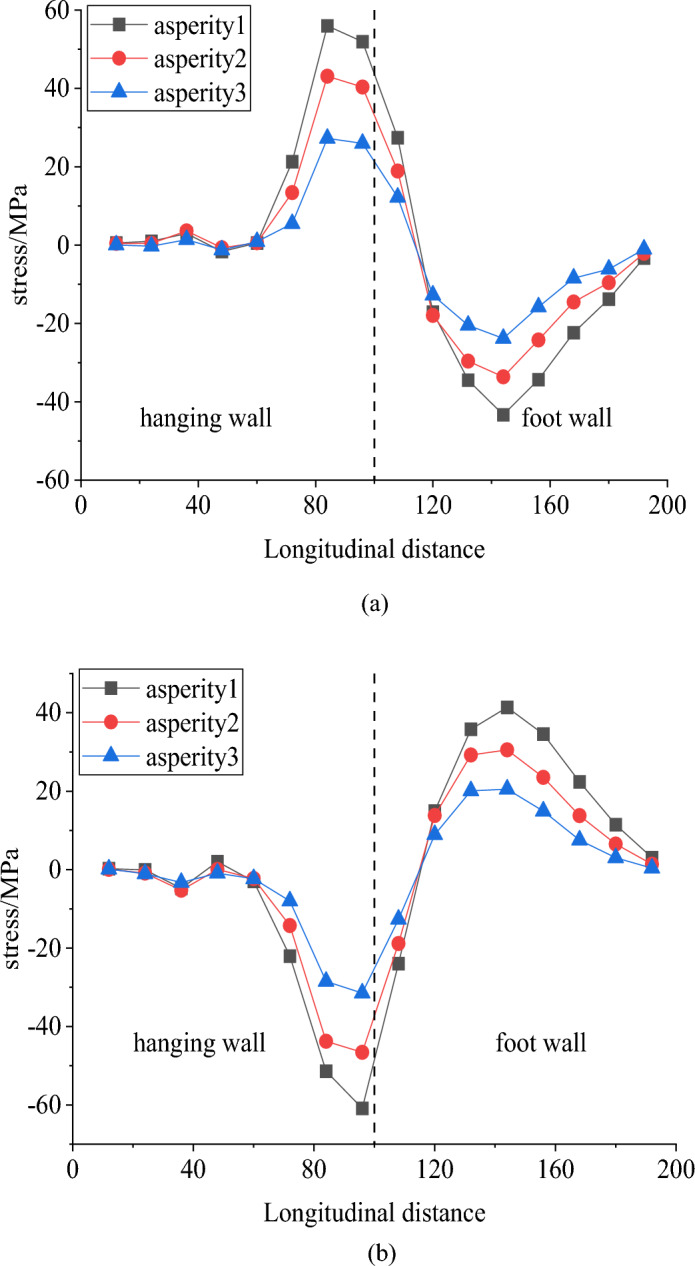
Longitudinal strain curve of tunnel under different asperities, (**a**) right side wall longitudinal stress and (**b**) left side wall longitudinal stress.

Figure 18 displays the longitudinal stress on the right and left side walls under three different asperity locations. As the asperity moves rightward along the fault, the displacement within the tunnel space decreases. According to Section "Effect of the fault mechanism on the tunnel", the focus is on the mechanical response of the side wall when the fault is left-lateral strike-slip. The results indicate that the peak longitudinal stress on both side walls decreases as the asperity location shifts to the right. Consequently, the closer the tunnel is to the large asperity, the higher the stress experienced by the tunnel.

## Conclusions

In this study, the seismic response of the Daliang tunnel due to the coseismic faulting of the 2022 M_s_6.9 Menyuan earthquake was analyzed using a fault-structure system. Our results and conclusions are summarized as follows:

1. To consider the heterogeneous distribution of slip on the fault plane, a nonuniform fault slip model was proposed. The Loma Prieta and 2022 Menyuan earthquakes were adopted to validate the nonuniform slip model. The simulation results show that the proposed nonuniform slip model achieves better performance than the uniform asperity model.
2. The seismic response of the Daliang tunnel due to the coseismic faulting of the Menyuan earthquake was analyzed using a discrete–continuous coupled numerical simulation. The field investigation revealed that the maximum dislocation of the tunnel exceeds 2.5 m. In contrast, the numerical model predicts displacements of 2.16 m and 2.17 m for the left and right side walls, respectively. The observed results satisfy the characteristics of left-lateral strike-slip faults. However, the simulated maximum dislocation was smaller than observed, especially vertically. This discrepancy is attributed to differences in rock parameters and the neglect of seismic wave effects in the model. Despite this, the simulation generally aligned with on-site seismic damage investigations. The findings of this study can provide valuable insights for the evaluation of the surrounding rock stability, as well as the design and construction of support structures for transverse fault tunnels.
3. The effects of different fault parameters on the seismic response of tunnels through an active fault were investigated using a discrete–continuous coupling numerical simulation. The numerical simulation results indicate that the distributions of the maximum stress and strain would change under different fault mechanisms. Fault slip can significantly affect the tunnel response. A tunnel through an active fault with a wider fault fracture zone and smaller dip angle would experience less damage.
4. Based on the above study, a fault-structure system can be employed to evaluate the seismic response of tunnels passing through active fault zones owing to coseismic faulting. The proposed nonuniform slip model is validated to obtain spatially varied ground and internal displacement due to fault slip. The discrete–continuous coupling numerical method can simulate the stress and strain characteristics of the lining under fault slips. For the seismic design of a tunnel through an active fault zone, the effects of different fault parameters on the response of the tunnels should be considered.

## Data Availability

All data generated or analysed during this study are included in this published article.
